# Lateral PbS Photovoltaic Devices for High Performance Infrared and Terahertz Photodetectors

**DOI:** 10.3390/nano11071692

**Published:** 2021-06-28

**Authors:** Emmanuel K. Ampadu, Jungdong Kim, Eunsoon Oh

**Affiliations:** Department of Physics, Chungnam National University, Daejeon 34134, Korea; ekampadu@cnu.ac.kr (E.K.A.); jungdong.kim@kepco.co.kr (J.K.)

**Keywords:** infrared photo-detector, photovoltaic, terahertz detector, photocurrent transient, FTIR

## Abstract

We fabricated a lateral photovoltaic device for use as infrared to terahertz (THz) detectors by chemically depositing PbS films on titanium substrates. We discussed the material properties of PbS films grown on glass with varying deposition conditions. PbS was deposited on Ti substrates and by taking advantage of the Ti/PbS Schottky junction, we discussed the photocurrent transients as well as the room temperature spectrum response measured by Fourier transform infrared (FTIR) spectrometer. Our photovoltaic PbS device operates at room temperature for wavelength ranges up to 50 µm, which is in the terahertz region, making the device highly applicable in many fields.

## 1. Introduction

Infrared (IR) photodetectors are widely applied with increasing importance in communications, environmental monitoring and security [1]. For infrared detection, various materials are extensively used, including Ge, InGaAs, InAs, and lead chalcogenides (PbTe, PbS and PbSe) [2]. Among these, PbS films are produced by various growth techniques, such as chemical bath deposition (CBD) [3,4,5,6], sputtering [7], electrodeposition [8], spray pyrolysis [9,10], microwave heating [11,12] and spin-cast of colloidal quantum dots [13,14]. Chemical bath deposition (CBD) is an efficient technique for the synthesis of high quality PbS thin films in laboratory conditions [15] as well as on large industrial scales [16]. The characteristics of chemically deposited PbS thin films by CBD strongly depend on the growth conditions and affect the device performance [3].

Commercialized PbS infrared detectors are of the photoconduction type, where external bias is applied to the devices [17,18]. Due to the narrow bandgap (~0.4 eV), the dark current background noise is expected to be relatively large, reducing the detectivity. On the other hand, photovoltaic PbS devices are developed with various heterojunctions [19,20,21] and with Ti/PbS Schottky junctions [22,23]. Photovoltaic devices which do not require any external bias have the advantages of negligible dark currents, low noise level and fast response. Both photoconduction and photovoltaic PbS devices operate for wavelength ranges up to 3–5 µm [24,25]. Moreover, photoconduction beyond 7 µm was also reported earlier using PbS, which was attributed to the carrier excitations from impurity levels [26,27].

Terahertz (THz) technology has applications in imaging, astronomy, biomedicine and security screening applications for detecting hidden objects [28,29,30]. THz radiation typically refers to the electromagnetic radiation in the frequency range from ~0.1 THz to 10 THz, corresponding to wavelengths from 30 μm up to 3 mm [30,31,32]. For the detection of THz radiation, various techniques have been employed, such as the use of bolometers which operate on the principle of change in resistance by the heat generated upon absorption [33]. However, these THz bolometers must be cooled down to low temperatures to achieve high thermal responsivities, greatly limiting their applications.

We reported earlier on Au/PbS/TiO_2_/FTO vertical photovoltaic devices and measured photo response up to the terahertz range [22]. In this paper, we show that metal/PbS/metal lateral photovoltaic detectors can be fabricated by simply depositing PbS directly on two metal electrodes. This lateral type detector does not require any top contact electrode, useful for the future development of infrared and terahertz detectors.

## 2. Materials and Methods

PbS films were grown by chemical bath deposition at room temperature. Prior to the growth, microscope slide glasses were cleaned with acetone, methanol, DI water and then dried with nitrogen gas. PbS films were deposited according to the procedures outlined in [34]. The resulting films were homogeneous and well adhered to the glass. For Hall measurements, PbS films were deposited on glass and Hall mobility was measured using Van der Pauw method. The surface morphology and the average thickness of PbS on both Ti and glass were determined by using a cold type field emission scanning electron microscope (FESEM, S-4800, Hitachi High-Technologies, Tokyo, Japan). The lateral photovoltaic device was fabricated by depositing PbS on Ti. The two Ti electrodes, separated by a gap of 7 mm, were deposited on glass by an e-beam evaporator using a metal mask. Photocurrent was measured with a 532 nm DSPP laser and a picoammeter connected to a computer. Photocurrent spectrum was recorded at 300 K with 0 V using a Bruker Vertex 80 V Fourier transform infrared (FTIR) spectrometer (Leipzig, Germany) equipped with a globar mid-infrared light source along with a KBr beam splitter. The modulation frequency used for the photocurrent measurement was 20 kHz.

## 3. Discussion

**Figure 1:**
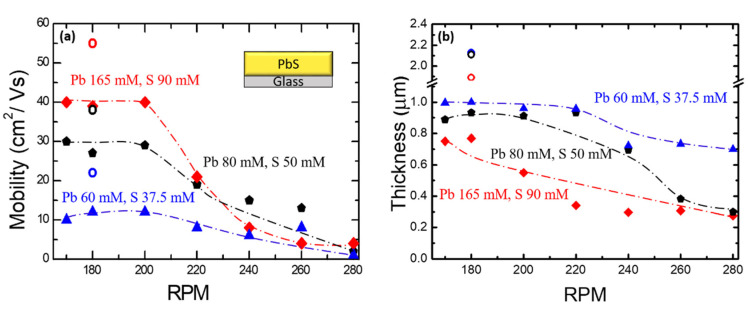
(**a**) Mobility and (**b**) thickness of PbS films as a function of RPM for various Pb and S concentrations. PbS film thicknesses were measured from cross-sectional SEM images. Both mobility and film thickness are seen to decrease as RPM increases. The dashed curves are for guiding eyes. Open circles (○) represent successive growth using the same concentrations.

From the Hall measurements, our PbS films were found to be p-type and the value of the hole concentrations varied between 2 × 10^17^ and 2 × 10^18^ cm^−3^. Figure 1a shows the mobility values of the PbS films deposited on glass as a function of stirring RPM (revolutions per min). The mobility values were observed to generally decrease as the stirring RPM was increased above 200, irrespective of the mole concentrations used. The chemical bath deposition of PbS has been described by the ion–by–ion deposition of precursors [35]. For an increased stirring RPM, the reaction rate of the ions increased and more precipitations were collected at the bottom of the Teflon beaker, rather than the precursor ions being deposited on glass substrates. The precipitations at the bottom of the Teflon beaker were more pronounced as mole concentrations increased. For larger RPM, the deposition rate on the substrates was decreased and the chemical reaction inside the solution was rather increased, which resulted in thinner films as shown in Figure 1b.

**Figure 2:**
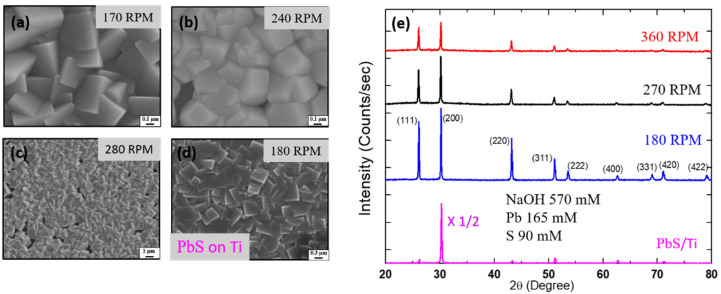
Surface SEM images of PbS films deposited on glass (**a**–**c**) and on Ti (**d**) using NaOH 570 mM, Pb 165 mM, S 90 mM. The values of stirring RPM are indicated. The density of the pinholes was increased as RPM was increased. (**e**) X-ray patterns of PbS deposited on Ti with 180 RPM and on glass with varying RPM values.

Figure 2 shows X-ray patterns and SEM images from PbS films with various RPM values for fixed mole concentrations. The peak intensity was found to increase with decreasing RPM. As seen from the SEM images in Figure 2a–c and Supplementary Figure S1a–i, the density of pinholes tends to increase with increasing RPM above 200 for three different mole concentrations. In order to obtain thicker films, we repeated the deposition on an already prepared film and continued the growth of PbS for an additional hour using a fresh solution of the same concentration. The mobility and thickness of the films (open circle, (○) thus obtained were found to be ~60 cm^2^/Vs and ~2 µm, respectively. The observed improvement of mobility (see Figure 1) after successive depositions was probably due to the reduced structural defect density as the films became thicker. The surface and cross-sectional images of successive depositions of the films obtained by varying the conditions are shown in Supplementary Figure S2a–f.

We fabricated a lateral type photovoltaic device which was made up of two Ti electrodes separated by a gap of 7 mm. We successively deposited PbS film by using NaOH 570 mM, Pb(NO_3_)_2_ 165 mM and CS(NH_2_)_2_ 90 mM all dissolved in 100 mL of DI water with 180 RPM. An X-ray pattern and an SEM image of a PbS film on Ti are included in Figure 2, where strong preferential growth along <100> is observed. The average crystallite size of the PbS film on Ti was estimated to be 1 μm in the vertical direction and 0.2 μm in the lateral direction [23]. The SEM images of a lateral device are shown in Supplementary Figure S3a–c. The shaded area of Supplementary Figure S3b represented an SEM image of the PbS film at the glass and Ti boundary. There seemed to be smaller and closely packed cubic microcrystals of PbS observed at the boundary.

**Figure 3:**
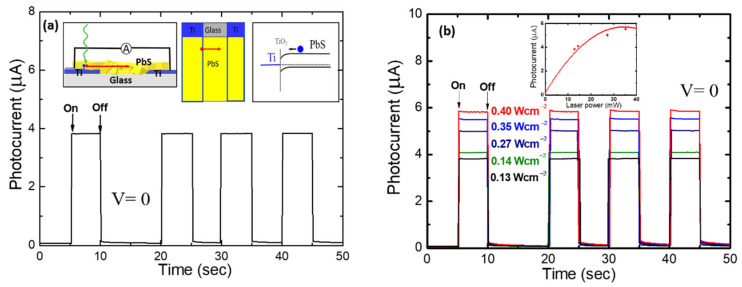
(**a**) Transient photocurrent characteristics of a lateral Ti/PbS/Ti photovoltaic device under the light excitation from a frequency doubled DSPP 532 nm laser. Schematics of the devices are included; the blue and red dots represent photo-electrons and photo-holes, respectively. The potential energy diagram of PbS and Ti Schottky junction is also shown. (**b**) Photocurrent transients for various excitation power densities. The inset shows the photocurrent saturation with respect to the excitation power.

PbS photoconduction devices suffer from high dark current noise, caused by an external bias. We pursued photovoltaic PbS devices for lower noise operation at zero bias. Photocurrent transients of our lateral type Ti/PbS/Ti device are shown in Figure 3. The excitation source was a 532 nm DSPP laser beam shone directly on the PbS film close to one of the Ti electrodes as shown in the top left inset of Figure 3a. The device exhibited both fast rise and fast decay components during on and off cycles of the laser beam. The fast rise was from the influence of the internal electric (drift) field due to the Schottky barrier formed between Ti and PbS (top right inset of Figure 3a). The Schottky barrier characteristics of Ti and PbS have been reported earlier [22,23,36,37]. As the laser was turned off, a sharp fall of the photocurrent transient was observed. Compared to the vertical devices described in [38], in this lateral structure, photo-generated holes travelled laterally to the opposite electrode in order to generate photocurrent. Owing to the high hole mobility obtained for CBD-PbS, photo generated holes were able to travel laterally to the other electrode.

Together with the rapid fall, slow decay components were also observed. The observed slow decay component is attributed to grain boundary defects which act as trapping sites. In PbS thin films grown by CBD, grain boundary defects serve as efficient electron trapping sites [39]. The relatively slow decay time represents the electron release time from the traps. Similar slow decay transients due to the profusion of grain boundaries making films more resistive, owing to nanometer-sized particles was observed in a PbS photoconductive device using a bias voltage of 1 V [15]. By careful observation, a small overshoot could be seen as the excitation power was increased. This overshoot was attributable to the accumulation of photoelectrons at the Ti/PbS interface [38]. Similar to the graphene (G)/PbS/Ti devices described in [38], it appears that more photo-electrons are accumulated at the Ti and PbS interface with increasing excitation power, which was evident from the photocurrent saturation (inset of Figure 2b).

We found that as the laser illumination spot was shifted to the center of the Ti electrode, the turn-on photocurrent transient was different from the transients described above as shown in Supplementary Figure S4. As seen, the magnitude of the photocurrent using the same laser power was decreased. This may be due to reduction in the efficiency of photo-carrier generation and transport [40]. As the laser was turned on and off, slow rise (slow decay) components accompanying fast rise (fast fall) were observed. The slow rise and slow decay transients may be due to trapping and de-trapping of photo-carriers in the PbS grain boundaries. We reported slow rise and slow decay transients in lateral photoconductive devices using PbS nanowires grown on Ti substrates, where the slow components were attributed to electron trapping and de-trapping in PbS nanowires [36].

**Figure 4:**
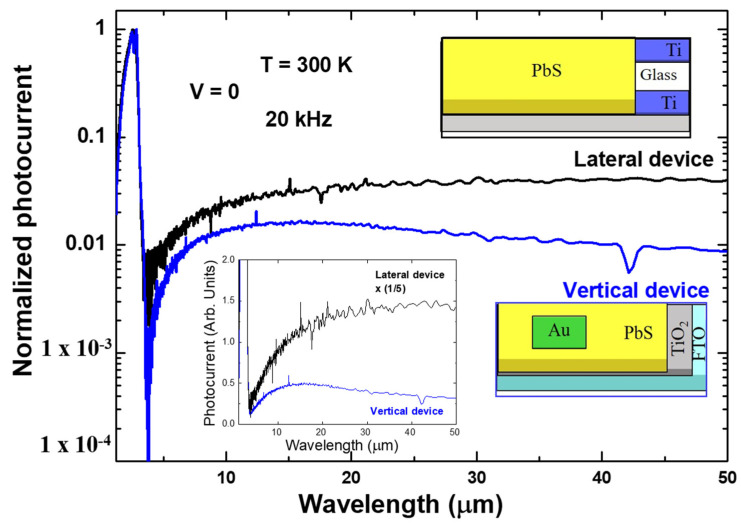
The normalized photocurrent spectrum of the lateral device (black) measured by an FTIR setup. Photocurrent spectrum of a vertical device is also shown for comparison. In order to emphasize the enhancement of the sub-bandgap response for the lateral device, the spectra are plotted in a linear scale, as shown in the inset.

Thanks to the fast response obtained from the lateral device, we were able to obtain a photocurrent response using an FTIR set-up. The photocurrent spectrum was recorded at 0 V and a modulation frequency of 20 kHz. Figure 4 shows the normalized photocurrent response at room temperature. The response for wavelengths shorter than 3.2 µm corresponds to the above-bandgap photocurrent. Surprisingly, for wavelengths above 20 µm, the photocurrent signal kept increasing with increasing wavelength. It was reported that at longer wavelengths the absorption coefficient from PbS was increased [41]. Sub-bandgap photoconductivity from PbS was also observed up to the order of 10 µm, which was attributed to the impurity band absorption [26,27]. From PbS vertical devices sub-bandgap photocurrent response was observed, where the maximum response was at around 15 µm [22]. The photocurrent spectrum from the vertical structure is included for comparison.

The increase of the photocurrent with increasing wavelength above 20 µm for our planar device indicates that photon energy as small as ~24 meV (50 µm) is responsible for a sub-bandgap excitation. We note here that two electron trapping levels known in CBD-grown p-type PbS films are 0.1 and 0.17–0.2 eV below the conduction band [42]. Thus, it is difficult to attribute the long wavelength photocurrent response to the excitation of electrons at these trapping sites. It is interesting to note that the photocurrent at such a long wavelength is significantly weaker when multilayer graphene (G) was used as a top contact electrode in a G/PbS/TiO_2_/FTO vertical structure (see Figure 5b in [22]), whereas the PbS top surface is completely exposed to the air in the planar device. Since the surface of PbS film is not at all protected by any passivation layer in our lateral device one may wonder whether the enhancement of the photocurrent at around 50 µm compared to other vertical structures is associated with the surface trapping.

Another possible origin is the excitation of the electrons accumulated at the PbS/Ti interface, where naturally oxidized layers are formed at the interface of Ti and PbS. We measured the photocurrent response up to 50 µm wavelength in a Au/PbS/TiO_2_/FTO vertical structure (see Supplementary Figure S5), where we employed an external green laser excitation through the FTO substrate [22]. It is not clear why the photocurrent response at such a long wavelength is enhanced in a lateral structure, but the lateral structure has an advantage that it can operate without the help of an extra light source, such as a green laser.

## 4. Conclusions

PbS films were chemically deposited by varying the mole concentrations and stirring RPM. Taking advantage of the Schottky contact of Ti/PbS, we fabricated lateral photovoltaic Ti/PbS/Ti devices. While most PbS lateral devices are the photoconduction type, photovoltaic type devices which do not require any external bias have the advantages of negligible dark currents and low noise level. Thanks to the rapid response, we were able to measure the photocurrent spectrum of the Ti/PbS/Ti lateral device using an FTIR set-up at 20 kHz. From the lateral device, we observed a strong photocurrent due to sub-bandgap excitations as compared to Au/PbS/TiO_2_/FTO vertical device. Our device can operate up to 50 µm which is in the terahertz region, making the device suitable for numerous applications.

## Data Availability

Publicly available datasets were analyzed in this study. This data can be found here: [https://drive.google.com/drive/folders/1gglvpUpqamC9MayCz4qHdv8qgorRU2Y7?usp=sharing].

## Supplementary Materials

The following are available online at https://www.mdpi.com/article/10.3390/nano11071692/s1, Figure S1: Surface SEM images of PbS films deposited on glass with various stirring RPM and mole concentrations, Figure S2: Surface and cross-sectional SEM images of PbS films deposited successively on glass, Figure S3: SEM images of PbS lateral devices, Figure S4: Time trace of photo response when the laser illumination spot was shifted to the center of the Ti electrode, Figure S5: Comparison of photocurrent spectra from vertical and lateral devices.
