# Supplementary material for: Lateral PbS Photovoltaic Devices for High Performance Infrared and Terahertz Photodetectors
Source: Nanomaterials (Basel). 2021 Jun 28;11(7):1692. doi: 10.3390/nano11071692 (PMC8306324; doi:10.3390/nano11071692)
Supplement: Supplementary file 1 [file nanomaterials-11-01692-s001.zip › nanomaterials-1256422-supplementary.pdf]

nanomaterials

Supplementary Materials

Lateral PbS Photovoltaic Devices for High Performance Infrared and Terahertz Photodetectors

Emmanuel K. Ampadu, Jungdong Kim and Eunsoon Oh *

Department of Physics, Chungnam National University, Daejeon 34134, Korea; ekampadu@cnu.ac.kr (E.K.A.); jungdong.kim@kepco.co.kr (J.K.)

* Correspondence: esoh@cnu.ac.kr

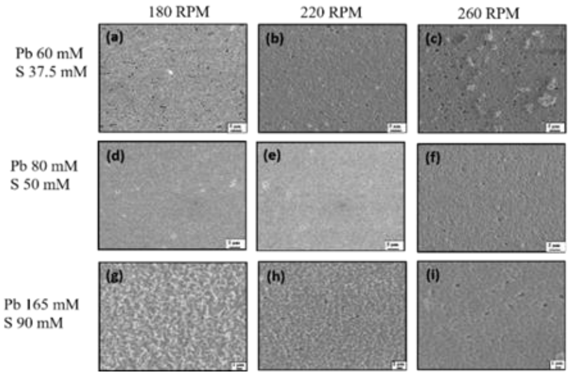

Figure S1. Surface SEM images of PbS films deposited on glass (**a**–**c**) Pb 60 mM, S 37.5 mM (**d**–**f**) Pb 80 mM, S 50 mM, (**g**–**i**) Pb 165 mM, S 90 mM. For all the depositions, NaOH concentration was 570 mM. The density of pinholes was increased as stirring RPM was increased.

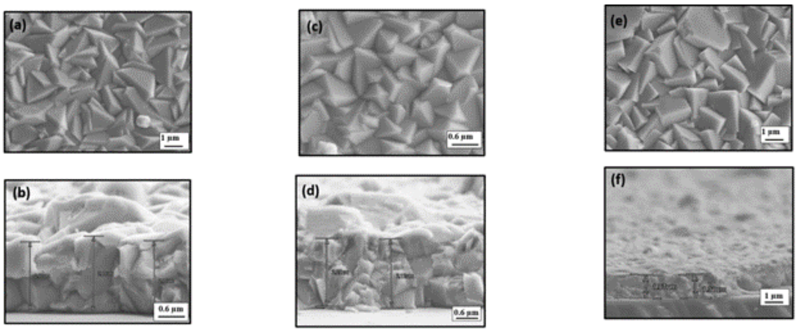

Figure S2. Surface and cross-sectional SEM images of PbS films deposited successively on glass (**a**,**b**) Pb 60 mM, S 37.5 mM (**c**,**d**) Pb 80 mM, S 50 mM, (**e**,**f**) Pb 165 mM, S 90 mM using 570 mM NaOH and 180 RPM.

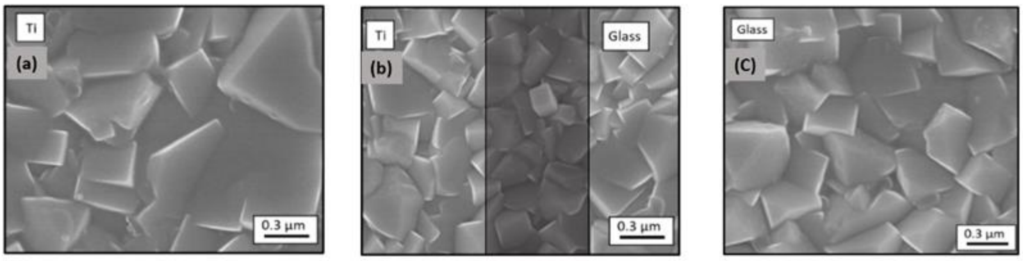

Figure S3. SEM images of PbS films deposited on the (**a**) Ti side and (**c**) glass side on a lateral device. Shaded area of (**b**) represents PbS growth at the Ti and glass boundary.

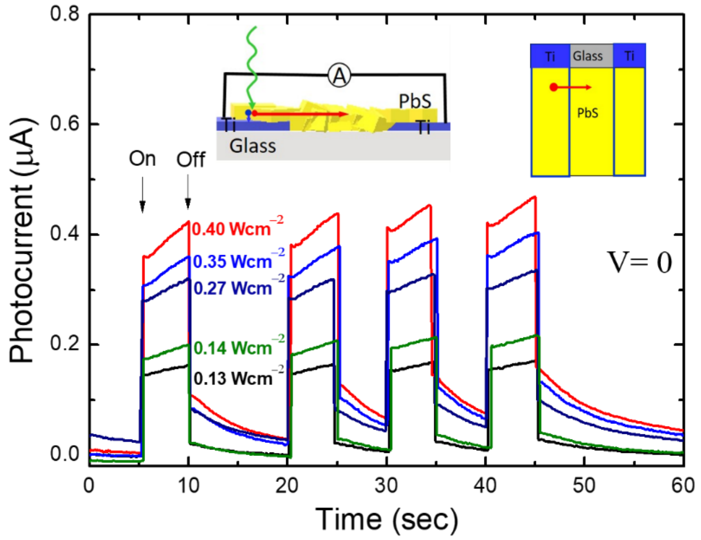

Figure S4. Photocurrent transient of a lateral Ti/PbS/Ti photovoltaic device. The laser spot was moved more toward the center of the Ti electrode. Accompanying the fast rise (fall) transients are slow rise (decay) components, which are attributed to the trapping and de-trapping of photo-carriers.

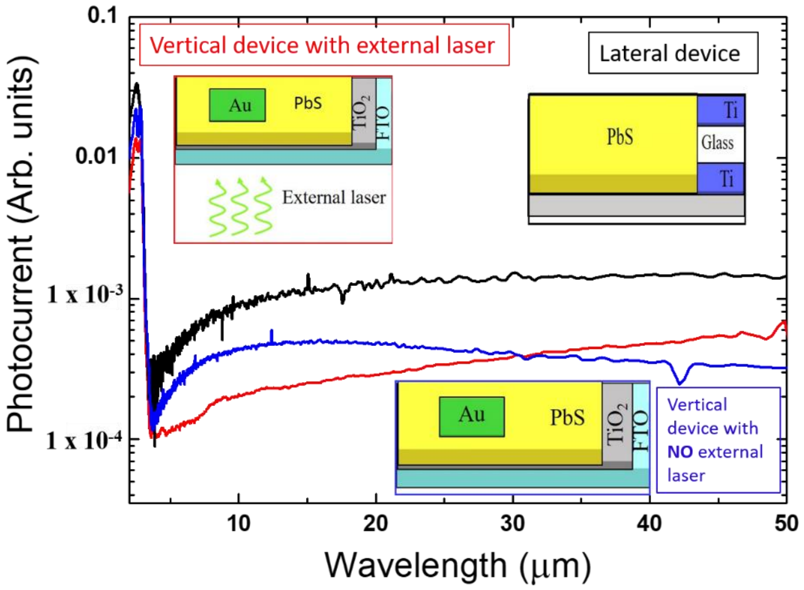

Figure S5. Room temperature photocurrent spectrum of a lateral device (red), a vertical device with an external backside illumination (blue) and a vertical device with no backside illumination (black). The lateral device has superior sub-bandgap photocurrent response as compared to the other two devices.
